# Unilateral Conjoint Tendon Formation of the Piriformis, Superior Gemellus, and Tendonized Obturator Internus: A Rare Cadaveric Variation of the Deep Gluteal Region

**DOI:** 10.7759/cureus.111829

**Published:** 2026-06-30

**Authors:** Bruno Palmieri, Brady Ryals, Lucas Norris, Claire Fourroux, Adegbenro O Fakoya

**Affiliations:** 1 Cellular Biology and Anatomy, Louisiana State University Health Sciences Center Shreveport, Shreveport, USA

**Keywords:** anatomical variation, deep gluteal region, obturator internus, piriformis muscle, sciatic nerve, superior gemellus

## Abstract

Anatomical variations of the deep gluteal musculature are uncommon but may have important implications for surgical approaches, radiologic interpretation, and the evaluation of posterior hip pain. During routine cadaveric dissection of an 89-year-old male donor, we identified a rare unilateral variation involving the deep external rotators of the right hip. The piriformis (Pirif.), superior gemellus (SG), and a markedly tendonized obturator internus (OI) converged to form a prominent common tendon inserting onto the greater trochanter. Although the inferior gemellus contributed to the common insertion, it did not substantially participate in the enlarged tendon, while the quadratus femoris (QF) maintained its typical independent insertion. Examination of the contralateral side demonstrated normal anatomy, confirming the unilateral nature of the variation.

The observed configuration expands the spectrum of known variations involving the deep external rotators of the hip. Embryologically, the anomaly may be explained by incomplete separation of the Pirif. and OI tendon complexes during development. The extensive tendonization of the OI and fusion with adjacent muscles may alter force transmission, hip stabilization, and external rotation mechanics. Furthermore, the close anatomical relationship between the fused tendon complex and the sciatic nerve suggests potential clinical relevance in deep gluteal syndrome, posterior hip pain, and sciatic nerve irritation.

This cadaveric case highlights the anatomical complexity and variability of the deep gluteal region and underscores the importance of recognizing such variants during anatomical dissection, surgical procedures, and radiologic evaluation. Awareness of these uncommon configurations may improve understanding of posterior hip pathology and aid clinicians in diagnosing and managing deep gluteal disorders.

## Introduction

The subgluteal region contains a complex arrangement of musculoskeletal and neurovascular structures that contribute to external rotation and stabilization of the hip joint. The deep gluteal musculature consists of the piriformis (Pirif.), superior gemellus (SG), inferior gemellus, obturator internus (OI), and quadratus femoris (QF) muscles [1]. Traversing this region are several clinically important neurovascular structures, including the sciatic (L4-S3), superior gluteal (L4-S1), inferior gluteal (L5-S2), pudendal (S2-S4), and posterior femoral cutaneous (S1-S3) nerves [2]. Given the close anatomical relationships among these structures, anatomical variations within the deep gluteal region may have important clinical and surgical implications.

The Pirif. muscle serves as a key anatomical landmark within the gluteal region. It originates from the anterior surface of the sacrum and the sacrotuberous ligament and inserts onto the superior border of the greater trochanter of the femur [3]. The OI muscle originates from the pelvic surface of the obturator membrane and the surrounding bony margins of the obturator foramen [3]. The SG arises from the ischial spine, whereas the inferior gemellus originates from the ischial tuberosity [3]. The tendons of the OI and the superior and inferior gemelli converge and insert onto the medial surface of the greater trochanter of the femur [3]. The QF originates from the lateral border of the ischial tuberosity and inserts onto the quadrate tubercle of the intertrochanteric crest of the femur [3].

During routine cadaveric dissection, we identified an unusual variation involving the Pirif., SG, and OI muscles. These structures converged to form a prominent common tendon inserting onto the greater trochanter. Although uncommon, variations involving the Pirif. and adjacent musculature have been reported in the literature. Fusion between the Pirif. and gluteus medius has been described, with the lower border of the Pirif. blending with the gluteus medius nearly to its insertion on the greater trochanter [4]. Associations between the Pirif. and SG have also been documented. In cases where the Pirif. presents with two muscle bellies, the tendon of the inferior belly may merge with the SG tendon before inserting onto the greater trochanter; this variation was reported in 5 of 511 specimens [4]. Additionally, conjoint Pirif.-OI tendons measuring between 0.5 and 2.0 cm have been described, although one study identified this arrangement in only 10% (3/29) of specimens [4].

While previously reported variations have demonstrated varying degrees of fusion involving the Pirif., SG, and OI muscles [4], the configuration observed in the present specimen appears to represent a more extensive and uncommon anatomical arrangement. This cadaveric case report describes a rare unilateral variation of the deep external rotators of the hip and highlights the anatomical complexity of the subgluteal region. Recognition of such variants is important for anatomists, surgeons, radiologists, and clinicians because of their potential implications for surgical approaches, imaging interpretation, and the evaluation of posterior hip pain and sciatic nerve-related disorders.

## Case presentation

**Figure 1:**
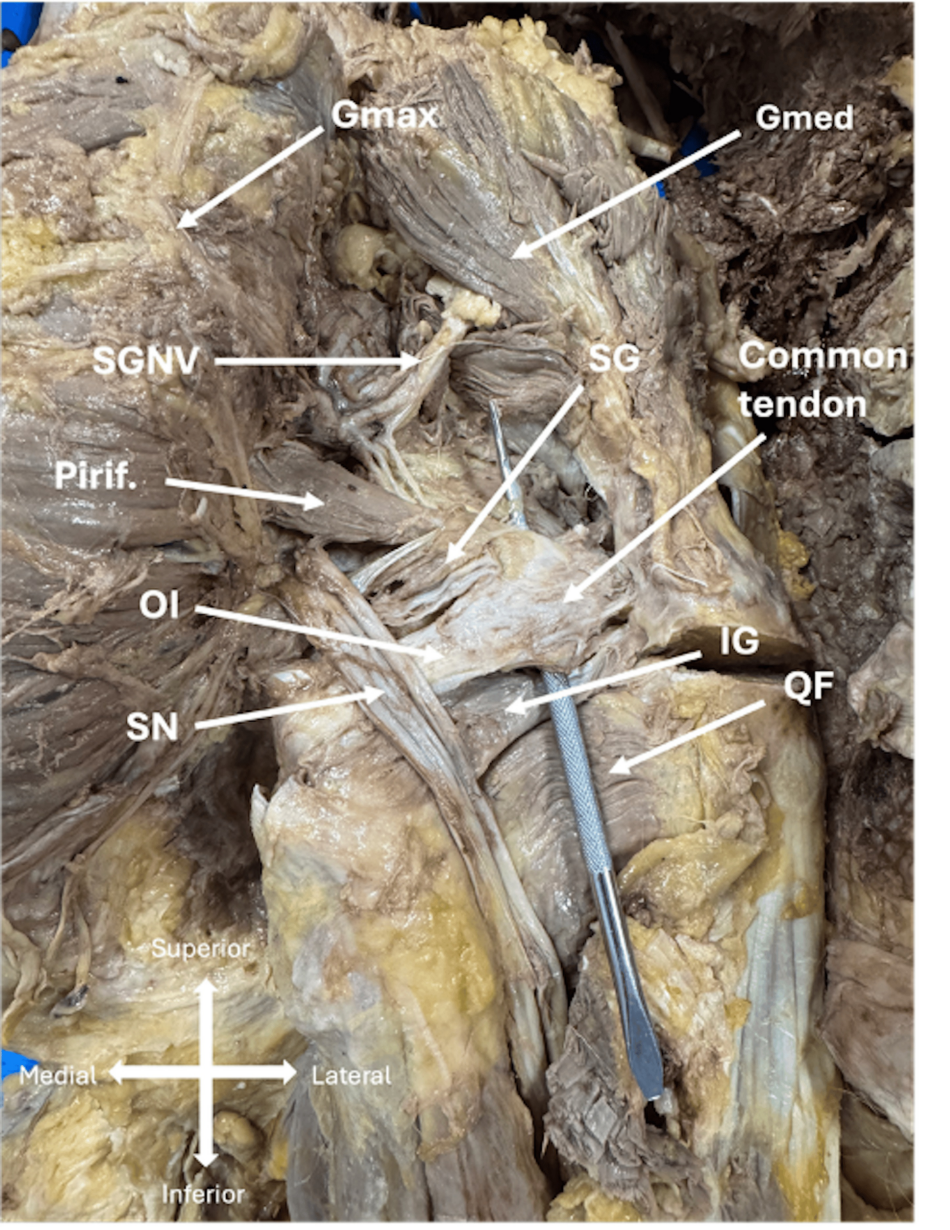
Rare Fusion of the Piriformis, Superior Gemellus, and Tendonized Obturator Internus Forming a Common Tendon in the Right Gluteal Region Posterior dissection of the right gluteal region demonstrating a rare variation of the deep external rotators of the hip. The piriformis (Pirif.), superior gemellus (SG), and a markedly tendonized obturator internus (OI) converge to form a prominent common tendon inserting onto the greater trochanter. The inferior gemellus (IG) joins the common insertion but contributes minimally to the enlarged tendinous complex. The quadratus femoris (QF) maintains its typical independent insertion and does not participate in the fusion. The gluteus maximus (Gmax) and gluteus medius (Gmed) exhibit normal morphology. The superior gluteal neurovasculature (SGNV) follows its expected course between the gluteus medius and minimus, while the sciatic nerve (SN) emerges inferior to the Pirif. and traverses the deep gluteal region in its usual anatomical relationship.

During routine cadaveric dissection at Louisiana State University Health Sciences Center in Shreveport, a rare anatomical variation involving the deep external rotators of the hip was identified in the right gluteal region of an 89-year-old male donor. The Pirif., SG, and OI muscles converged to form a prominent common tendon that inserted onto the greater trochanter of the femur (Figure 1). The enlarged morphology of this tendon was primarily attributable to the OI, which was extensively tendonized before merging with the common tendinous structure. Although the inferior gemellus (IG) also inserts into the common tendon, its contribution to the enlarged superior tendon was comparatively limited (Figure 1). The QF maintained its typical anatomical configuration and inserted independently of the fused tendon complex.

This arrangement represents an unusual variation involving the short external rotators of the hip. Additionally, the sciatic nerve (SN) emerged inferior to the Pirif. and followed its normal course superficial to the deep external rotators without evidence of an aberrant relationship to the fused tendon complex (Figure 1).

**Figure 2:**
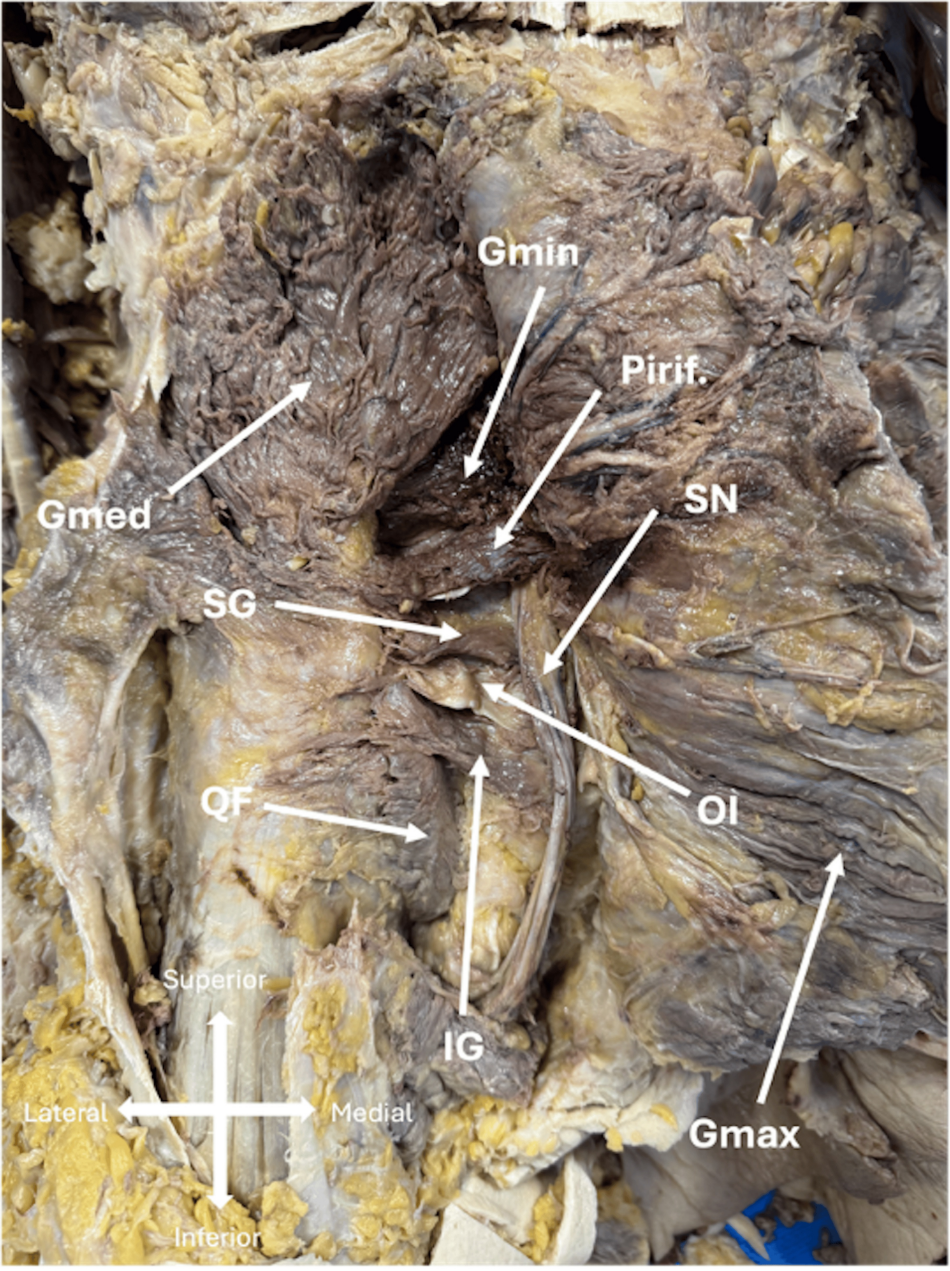
Contralateral Left Gluteal Region Demonstrating Typical Anatomy and Absence of Tendinous Fusion Posterior dissection of the left gluteal region demonstrating the typical anatomical arrangement of the deep external rotators of the hip. The piriformis (Pirif.), superior gemellus (SG), obturator internus (OI), inferior gemellus (IG), and quadratus femoris (QF) exhibit their expected morphology and anatomical relationships. The sciatic nerve (SN) emerges inferior to the Pirif. and follows its usual course through the deep gluteal region. The gluteus maximus (Gmax), gluteus medius (Gmed), and gluteus minimus (Gmin) display normal morphology.

Examination of the contralateral (left) gluteal region revealed the expected anatomical arrangement of the deep external rotators (Figure 2). Although the left OI also demonstrated a degree of tendonization before insertion, there was no evidence of significant fusion with adjacent musculature. The Pirif., SG, IG, and QF displayed normal morphology and insertion patterns, with each structure maintaining its expected anatomical relationships. The marked asymmetry between the right and left gluteal regions confirms that the observed tendinous fusion was unilateral rather than bilateral.

## Discussion

The OI is a triangular muscle that originates from the pelvic surface of the obturator membrane and adjacent osseous structures before inserting onto the medial surface of the greater trochanter of the femur [5]. As its fibers course posteriorly, they converge to form a tendon that passes through the lesser sciatic foramen before attaching to the femur. Owing to its anatomical position and attachments, the OI serves as an important functional link between the pelvis and hip joint. Together with the Pirif., SG, IG, and QF, it forms the deepest muscular layer of the gluteal region and contribute to external rotation and stabilization of the hip [5]. As one of the short external rotators, it functions in opposition to the primary medial rotators of the hip, including the gluteus medius, gluteus minimus, tensor fasciae latae, and portions of the adductor musculature [5].

Although external rotation of the femur is its primary action, the OI also contributes to abduction of the flexed hip, a function that complements the role of the obturator externus in controlling hip motion [5]. In addition to its role in movement, the OI is an important dynamic stabilizer of the hip joint. Biomechanical investigations have demonstrated muscle shortening during loaded transitions from hip flexion to extension, suggesting an important contribution to activities such as ambulation, stair climbing, and rising from a seated position [6]. Consequently, the OI is frequently considered in gait analysis, rehabilitation strategies, and studies evaluating functional recovery following hip pathology [6]. Clinically, the muscle may also be affected during posterior hip dislocations, in which injury to the short external rotators and surrounding gluteal musculature is common [5].

Despite its functional importance, anatomical variations involving the OI are infrequently reported. Previous studies have documented age-related increases in fibrosis and reductions in force-generating capacity of the OI in females over 60 years of age [7]. However, the etiology and clinical significance of structural alterations involving this muscle remain incompletely understood [7].

Embryological development provides a plausible explanation for the variation observed in the present specimen. During early fetal development, the Pirif. tendon is initially guided toward its insertion by the OI tendon before becoming separated and subsequently incorporated into the gluteus medius tendon complex [8]. The gluteus medius then directs the Pirif. toward its final insertion, while the superior and inferior gemelli facilitate separation of the OI from the developing tendon complex [8]. Disruption of this developmental sequence may explain the extensive fusion observed in the present case. Specifically, the incomplete separation of the Pirif. and OI tendon complexes during development may have resulted in persistence of a common tendinous structure extending into adulthood.

Although previous reports have described varying degrees of fusion between the Pirif. and OI tendons [9-13], the present specimen demonstrates a more extensive anatomical configuration characterized by marked tendonization of the OI and incorporation of the Pirif. into the conjoint tendon shared by the SG, OI, and inferior gemellus. While associations between these tendons have been previously documented [9-13], the extent of tendonization and fusion observed in this specimen appears unusual and expands the spectrum of known variations involving the deep external rotators of the hip.

The extensive tendonization of the OI may have biomechanical implications. Tendons generally facilitate efficient transmission of force, whereas muscle tissue contributes to active contraction, modulation of force, and dynamic control of movement [14,15]. Consequently, replacement of contractile tissue with a larger tendinous component may alter the balance between force transmission and fine motor control. Furthermore, incorporation of the Pirif. into the common tendon may reduce its ability to function independently as an external rotator and abductor of the hip. Although the functional consequences of this variation cannot be determined in a cadaveric specimen, it is reasonable to hypothesize that alterations in hip stabilization, force modulation, and external rotation mechanics may occur in individuals exhibiting a similar anatomy.

The unilateral nature of this variation is also noteworthy. Examination of the contralateral side revealed a comparatively typical arrangement of the deep external rotators, suggesting that the developmental process occurred independently on the affected side. Such asymmetry raises the possibility of differences in force transmission and load distribution across the hips. While limb dominance has been shown to influence gait mechanics and lower-extremity function [16], the absence of clinical history precludes determining whether the observed variation contributed to functional asymmetry during life.

Variations involving the Pirif. muscle have long been associated with sciatic nerve irritation and piriformis syndrome [17]. More recently, attention has shifted toward the broader concept of deep gluteal syndrome, in which multiple structures of the deep gluteal space, including the OI-gemelli complex, may contribute to posterior hip pain and sciatic nerve entrapment [18,19]. In the present case, the extensive fusion and tendonization of the Pirif.-OI complex may have increased regional stiffness and reduced the capacity of the surrounding musculature to accommodate sciatic nerve excursion during hip motion [20]. Although speculative, such an arrangement could theoretically predispose an individual to sciatic nerve irritation, deep gluteal pain, or symptoms resembling piriformis syndrome.

Collectively, these findings emphasize the importance of recognizing variations of the deep gluteal musculature. Awareness of such anatomical configurations is relevant not only to anatomists but also to surgeons, radiologists, physical therapists, and clinicians involved in the evaluation and management of posterior hip pain, deep gluteal syndrome, and disorders affecting the sciatic nerve.

## Conclusions

This cadaveric case report describes a rare unilateral variation of the deep gluteal region characterized by fusion of the Pirif., SG, and a markedly tendonized OI into a prominent common tendon inserting onto the greater trochanter. Although variations involving the Pirif. and OI have been previously reported, the extent of tendonization and fusion observed in this specimen appears uncommon and expands the spectrum of known anatomical variations of the deep external rotators of the hip.

The observed configuration may be attributable to incomplete separation of the Pirif. and OI tendon complexes during embryologic development. While the functional consequences cannot be determined from a cadaveric specimen, the altered musculotendinous architecture may influence hip biomechanics, force transmission, and dynamic stabilization. Furthermore, given the close anatomical relationship between the deep external rotators and the sciatic nerve, such variations may have implications for deep gluteal syndrome, posterior hip pain, and sciatic nerve-related symptoms.

Recognition of uncommon anatomical variants within the deep gluteal region is important for anatomists, surgeons, radiologists, and rehabilitation specialists. Documentation of these variations contributes to a more comprehensive understanding of clinically relevant anatomy and may assist in interpreting imaging studies, planning surgery, and evaluating patients presenting with posterior hip and gluteal pain syndromes.
